# An Insight into Testicular Macrocalcification—A Retrospective Study of 42 Cases on a Rare Sonographic Finding

**DOI:** 10.3390/life15020205

**Published:** 2025-01-30

**Authors:** Malene Roland Vils Pedersen, Ditte Marie Toft, Jan Lindebjerg, Søren Rafael Rafaelsen, Søren Kissow Lildal

**Affiliations:** 1 Department of Radiology, Lillebaelt Hospital, Beriderbakken 4, 7100 Vejle, Denmark; 2 Department of Regional Health, University of Southern Denmark, Campus 55, 5230 Odense M, Denmark; 3 Discipline of Medical Imaging and Radiation Therapy, School of Medicine, University College Cork, T12 AK54 Cork, Ireland; 4 Department of Pathology, Vejle Hospital, University Hospital of Southern Denmark, 7100 Vejle, Denmark; 5 Department of Urology, Urological Research Centre, Lillebaelt Hospital, Beriderbakken 4, 7100 Vejle, Denmark

**Keywords:** macrocalcification, microcalcification, testicular cancer, ultrasound

## Abstract

A single testicular microlithiasis is a common finding during sonography, while macrocalcification is a rare and incidental finding. The literature on macrocalcification is limited. Typically, testicular calcifications, whether microscopic or macroscopic, are benign but they can have a clinical significance. This multicenter study aimed to investigate the symptoms and prevalence of testicular cancer in patients with macrocalcification. Methods: Testicular ultrasound examination reports from four hospitals’ PACS database, covering the period 2014–2023, were screened for diagnoses of macrocalcification. Inclusion criteria required that the radiology report described macrocalcification supported by ultrasound images. Results: Macrocalcifications were identified in 42 male patients, with a mean age of 45 years. Sixteen macrocalcifications were in the right testicle, twenty in the left, and six were bilateral. Microlithiasis were found in 22 patients (52.4%), with 11 (26.2%) bilateral, 3 (7.1%) left-sided, and 8 (19.1%) right-sided. Testicular tumors were found in six patients. Conclusion: Testicular macrocalcification exhibited large visual variation and diverse clinical history. However, we found a low number of patients with testicular macrocalcification and testicular tumors, indicating that macrocalcifications have a benign nature, and that macrocalcification alone should not be a primary concern for malignancy, but this needs to be confirmed in further studies.

## 1. Introduction

Calcifications within the testicles have been documented as testicular microlithiasis, macrocalcification, or calcinosis, and are seen as a deposition of calcium. Testicular microlithiasis, with a size of 1–2 mm, is the most documented condition and is visualized intratesticularly by ultrasound [1,2,3]. Macrocalcification can be observed both extratesticularly and intratesticularly and is typically ≥3 mm in size [4,5]. On the other hand, calcinosis is the rarest condition, seen as calculi deposits in the scrotal layers of the tunica vaginalis dermis, often presenting as solitary nodules on the scrotal skin [6,7]; they typically have no clinical relevance, but can affect the patient’s quality of life due to their appearance directly on the scrotal skin.

Macrocalcifications are typically diagnosed by ultrasound and are seen as a round or oval-shaped hyperechoic area, often appearing as irregular. In biopsy and orchiectomy specimens, two types of calcifications have been identified in microlithiasis, hematoxylin bodies and laminated calcifications [8] located in the seminiferous tubules. However, the classification of macrocalcifications appears to be more complex to categorize. Another distinction between macrocalcification and microlithiasis is that the number of microlithiasis has been reported to fluctuate during childhood, with both increases and decreases observed [9]. Necas et al. observed that seminoma tumors with calcification had a larger average size (52 mm) compared to those without calcification (average size = 39 mm). However, the study did not specify the type of calcification observed in a small cohort [10].

The prevalence of macrocalcifications in unreported. Lotti et al. investigated 248 healthy fertile men with color Doppler ultrasound and found 1.2% with macrocalcifications [11].

The origin of macrocalcification remains unclear, and only speculations have been proposed based on specific populations such as chronic scrotal microtrauma observed in mountain bikers [12]. Also, suggestions regarding an association with hydrocele [13], infertility [14], and macrocalcifications being secondary to inflammations [15] have been proposed, although with limited data. Additionally, macrocalcification has been observed in patients with burn-out tumors [15]. A study presented six patients with macrocalcification in two hospital settings, who later developed a testicular cancer during follow-up [4].

Ultrasound is the first choice of modality due to its dynamic, real-time capabilities, and high-resolution imaging, including being a rapid and harmless diagnostic tool, even though it can be operator-dependent. In clinical practice, operator dependence in assessment and interpreting may seem limited during testicular ultrasound examinations. One strength of ultrasound is that it has the ability to detect testicular alterations in size and echotexture.

Still, studies have shown that observer variation is low during testicular ultrasound imaging [16,17]. In general, calcifications are considered a benign condition; however, microlithiasis and macrocalcifications can be observed in testicular cancer patients [3,5,15,18,19,20,21].

Current guidelines for testicular calcification address how to manage findings of microlithiasis. Most often, it is encouraged that macrocalcifications are included in the radiology report if their size is > 3 mm. There are limited guidelines available for patients with macrocalcifications, and most end with speculatives discussions about a potential risk of malignancy [22,23]. There is a discrepancy in the existing literature as other authors claim that macrocalcifications have never worried clinicians or sonographers. In general, limited evidence exists on this subject. An association between microlithiasis and testicular cancer in infertile men has been found in a systematic review including case–control studies [24]; however, microlithiasis has not been reported as an independent risk factor [2,25,26].

In this study, we present patients with macrocalcifications from a large regional hospital database from the Region of Southern Denmark. The study’s purpose was to investigate symptoms related to macrocalcifications and the prevalence of testicular cancer.

## 2. Materials and Methods

Ethics approval to report this retrospective study was sought at the local hospital board (17/30640) who waivered the need for informed consent. All the patients had a standard scrotal ultrasound examination performed for a variety of clinical reasons.

### 2.1. Patient Population

Denmark is divided into 5 local regions, all with one large university hospital and several medium or small hospitals, e.g., the Region of Southern Denmark has Odense university hospital and 4 medium hospitals. The population of the region of southern Denmark is 1.2 million [27].

A retrospective study investigated all data from the Region of Southern Denmark’s Picture Archive Communication System (PACS) testicular ultrasound investigation during an 18-year period (2006 to 2023). The cohort included patients diagnosed with testicular macrocalcification from four hospitals (Vejle, Kolding, Frederica, and Middelfart hospitals). The data search for scrotum investigation in the regional PACS was performed by a data manager system expert and included specific terms such as macrocalcification or calcification to identify relevant patients.

### 2.2. Data Collection

Each patient is assigned a unique personal identification number, called central person register (CPR) number. This unique identification number is used in all public national registries, enabling an individual-level correspondence between data and registries [28,29]. The CPR number was used to collect information about testicular biopsies and cancer subtypes when relevant. Data included the number of follow-up scans, pathology findings including testicular cancer subtype, the presence of testicular microlithiasis, and the location and numbers of macrocalcifications. All ultrasound images was assessed for confirmation of macrocalcifications.

Descriptive statistics were used, and a chi-squared test was performed to determine whether there a significant association between calcifications and testicular conditions. A *p* value equal or below 0.05 was considered statistically significant.

### 2.3. Scan Procedure

The testicular ultrasound scans were performed by either radiologists or senior sonographers. The database included information on hypo- and hyperechogenic lesions, the size of calcification, the number of follow-up scans, patient age, and tumors. The patients were referred to one of the four radiology departments by their local general practitioner.

All scans were performed on both testicles and were assessed by b-mode longitudinal and transverse planes comparing the two. No contrast agents were applied, as they are not implemented in any of the departments. Color Doppler was applied when appropriate. All data were reviewed by one of the authors (observer DMT), and in cases of uncertainty, the images were reviewed by a second author (MRP). The patients’ first examination served as the baseline. Macrocalcification was defined as ≥3 mm in size; if multiple, all were measured.

### 2.4. Ultrasound Technique

All ultrasound examinations were performed with high-frequency linear array probes using a variety of ultrasound machines (Simens S2000/S3000 (Acuson Corp, Mountain View, CA, USA); Hitachi EUB-8500 (Tokyo, Japan); Hitachi HI VISION Ascendus (Hitachi Medical Systems GmbH, Wiesbaden, Germany). All examinations were performed according to local hospital protocol, with radiology and ultrasound images stored in the Picture Archive Communication System shared by all 4 departments.

## 3. Results

A total of 33,137 scrotal examinations were performed between 2014 and 2023 in the Region of Southern Denmark, and a total of 95 patients were diagnosed with macrocalcification. Macrocalcification prevalence in the Region of Southern Denmark was 0.28% in a symptomatic population, in an area with approximately 600,000 males.

We obtained permission to investigate macrocalcification across four hospitals in the Region of Southern Denmark. In the four hospitals, we identified 42 patients and of those, 20 (47.6%) had bilateral macrocalcification. A total of four patients had undergone vasectomy prior to macrocalcification diagnosis.

The 42 patients ranged in age from 17 to 89 years (mean 45.6 years). See patient characteristics detailed in Table 1. Two patients were diagnosed with bladder cancer (one prior and one after macrocalcification diagnosis). Two were later diagnosed with colorectal cancer, and one with prostate cancer. One patient had the right testicle removed due to macrocalcification, but no tumor was detected, and the left testicle biopsy was normal.

Macrocalcification ranged from 3 to 8 mm in size. Follow-up was performed in 11 (26.2%) patients. A total of 13 (31%) of the 42 patients had previously undergone a scrotal ultrasound scan without macrocalcification being reported. Testicular tumors confirmed by histopathology were found in six (14.3%) patients.

**Figure 1:**
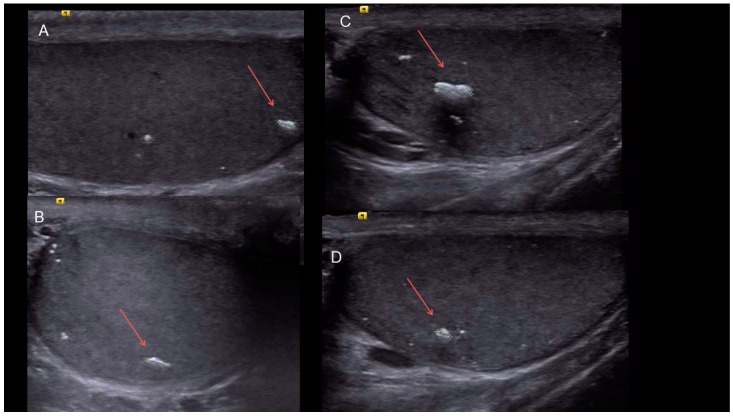
The four images show the macrocalcifications visualized in ultrasound images in a 51-year-old patient with bilateral macrocalcifications and no malignancy. (**A**,**B**) show the right testicle, and (**C**,**D**) show the left testicle with limited microlithiasis and macrocalcifications.

**Figure 2:**
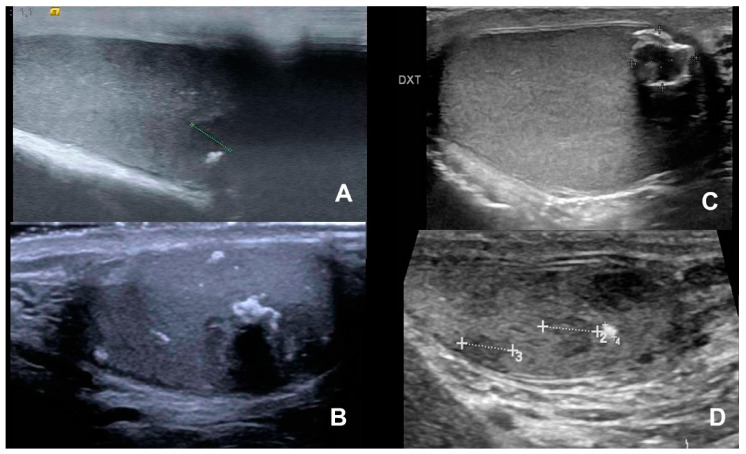
Variation in macrocalcifications visualized in patients with testicular cancer. (**A**) shows macrocalcification with mixed tumor, (**B**,**C**) show macrocalcification with embryonal carcinoma, and (**D**) shows macrocalcification in a seminoma.

**Figure 3:**
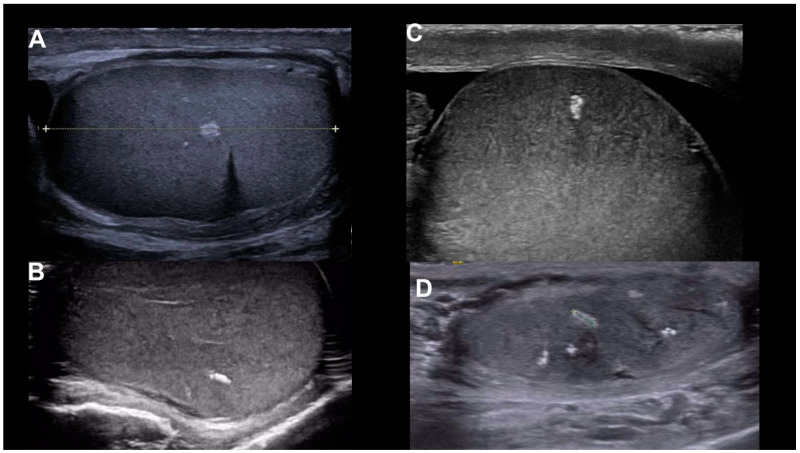
(**A**–**D**) show the variation in macrocalcifications in patients without any malignancy.

Figure 1 shows bilateral macrocalcifications in a 51-year-old male, whereas Figure 2 shows macrocalcification in relation to malignancy, and Figure 3 a variation without any malignancy.

All the patients were referred by their general practitioner based on scrotal pain (n = 17), scrotal welling, enlargement, tenderness, or discomfort (n = 15), nodule (n = 8), and trauma or hemospermia (n = 2). Among the 36 patients without testicular tumor detection, none have developed testicular malignancies as of August 2024.

Table 2 shows clinical findings based on ultrasound investigations on an individual level.

The chi-squared test showed no significant difference between macrocalcification compared to microlithiasis in relation to malignancy (*p* = 0.158), spermatocele (*p* = 0.367), hydrocele (*p* = 0.424), or varicocele (*p* = 0.119).

## 4. Discussion

We found a prevalence of 0.28% in a symptomatic population in the Region of Southern Denmark; however, the true prevalence in Denmark remains unknown. Since limited data exist, we speculate that this is a low prevalence, but more studies are warranted for confirmation.

We found a total of 42 patients with macrocalcifications, of which 6 (14.3%) had bilateral findings. Bilateral macrocalcifications in patients seem to be a rare finding. To the best of our knowledge, no other study exists on bilateral macrocalcifications. On the other hand, bilateral testicular microlithiasis has been reported in numerous studies [1,21,23] and is a common finding. Only two of the patents had bilateral macrocalcification and bilateral microlithiasis.

The molecular mechanism of macrocalcification is important to understand because of the impact of the condition. Despite this relevance, no studies have been published on its pathogenesis, to the best of the authors’ knowledge. Factors such as inflammation, trauma, and infertility could contribute to development, but this is currently mostly speculation. More clinical studies could help understand the underlying mechanisms and development.

It has been suggested that hydrocele and macrocalcification are associated; however, we observed hydrocele in 8 (19%), varicocele in 12 (28.6%), and spermatocele in 14 (33.3%) patients. Hydrocele, spermatocele, and varicocele are all common testicular conditions and an association with macrocalcification seems highly unlikely. We detected six patients with a testicular tumor. This dataset does not indicate a direct association between macrocalcifications and a risk of testicular cancer. However, more studies are warranted to explore whether macrocalcifications could potentially increase the risk of testicular cancer.

Limited data exist about macrocalcification and follow-up programs. The existing literature on ultrasound follow-up is sparse, with only limited studies addressing the long-term monitoring of patients with macrocalcification and microlithiasis. One study reported follow-up in two patients: one patient had an ultrasound scan six months after the initial diagnosis and then continued to clinical follow-up, and the other patient had a total of six follow-up ultrasound scans with a six-month interval, with no observed change or growth in the calcification in the follow-up period [24]. Without more evidence, it remains unclear what the best practices are for monitoring these patients in clinical practice.

Some studies have observed testicular microlithiasis and infertility or testicular dysfunction [30], but not all have found this association [31]. A recent study investigated 167 biopsies from a Caucasian population with microlithiasis and found 23.4% (n = 36) to have low semen quality [32]; this is supported by a review that found that microlithiasis is associated with decreased semen parameters and sperm concentration [33]. Presently, it is unknown whether infertility is seen in male patients with macrocalcifications.

There is a variation in the size of the reported macrocalcifications. For example, Bardisi et al. documented a 9 mm macrocalcification in a 28-year-old patient [34]. Similarly, Floranovic et al. identified a 10 mm macrocalcification in a 24-year-old male with a large-cell calcifying Sertoli tumor [35]. Other studies have reported smaller macrocalcifications: Taso et al. found a 5 mm left-sided macrocalcification [36], and Gurioli et al. reported a 4 mm calcification located in the upper testicular pole in a patient diagnosed with seminoma [37]. Additionally, Peroux et al. reported on an 18-year-old male with two macrocalcifications in the right testis [38]. Also, Deganello et al. found macrocalcification in two cases, representing 0.4% of the study population [39]. Desmousseaux et al. reported a higher prevalence, with 17 (35%) out of 48 patients having macrocalcifications in a population with malignant lesions [40]. Our findings align with the existing literature, showing a range of macrocalcifications between 4 and 8 mm in size, which is consistent with the sizes reported in other studies.

The main limitation is this study’s retrospective design, as the ultrasound machines will typically change over time, albeit typically becoming better. It is possible that we have missed some patients with macrocalcifications if the word calcification or macrocalcification was not written in the radiology report, and thereby introduced selection bias. Also, variations in populations or imaging practices may exist from country to country, adding another layer of complexity and making it challenging to draw conclusions. Further research is warranted to establish the prevalence of macrocalcifications and whether there is an association to testicular cancer or other pathologies.

This is the largest cohort study including macrocalcifications to the best of the authors’ knowledge. Due to the retrospective study design, investigations into extremely rare clinical conditions can be relatively easily conducted. It is a strength that these data offer clinical information combined with ultrasound images for clinicians to have a more profound understanding of this condition. In a previous study, we investigated three patients with macrocalcifications [4], but as all of those patients had macrocalcifications below 3 mm, they were excluded from this study.

## 5. Conclusions

Limited evidence exists about the association between testicular macrocalcifications and testicular cancer. This retrospective study shows macrocalcification prevalence to be low. It is likely that in the six cases of testicular cancer, the presence of macrocalcification is seen by chance, without any true association.

The practical implications of this study suggest that follow-up for testicular cancer in patients diagnosed with macrocalcifications may not be necessary based on our findings. However, clinicians should always consider individual patient risk factors when making follow-up decisions.

## Figures and Tables

**Table 1:** Patient characteristics.

Macrocalcification	N = 42	(%)
Left	20	(47.6)
Right	16	(38.1)
Bilat	6	(14.3)
Microlithiasis (n = 22)		
Left	3	(13.6%)
Right	8	(36.4%)
Bilat	11	(50.0%)
Testicular tumors (n = 6)		
Seminoma	2	(33.3%)
Non-Seminoma	4	(66.7%)

**Table 2:** Clinical findings in the 42 patients.

Id	Patient Age	Macrocalcifications	Side	Microlithiasis	Testicular<Cancer	Other <Findings
1	41	1	Left	Bilat	No	Spermatocele
2	47	4	Left	Right	No	Bilat hydrocele
3	38	10	Right	Bilat	No	Varicocele (left)
4	73	1	Left	Bilat	No	Varicocele (left)
5	51	Numerous	Bilat	Bilat	No	Varicocele (left)
6	37	1	Right	-	No	Spermatocele (left)
7	57	1	Left	-	No	Bilat varicocele, bilat spermatocele, lesion
8	27	Numerous	Bilat	Bilat	No	Hyperechoic area 5 mm
9	36	1	Left	-	Mixed tumor ^1^	Bilat hydrocele, tumor
10	39	Numerous	Left	Left	Seminoma	Varicocele (left), tumor (left)
11	67	1	Left	Right	No <(biopsy normal)	Tumor (left)
12	38	Few	Left	-	No	Funiculitis/inflammation of spermatic cord
13	24	Few	Bilat	-	No	Spermatocele (right)
14	54	1	Left	-	No	Sequelae recent epididymitis
15	36	1	Right	Bilat	No	Bilat spermatocele
16	65	Few	Right	Right	No	Bilat spermatocele, varicocele (left)
17	89	1	Right	-	No	Bilat spermatocele, bilat varicocele
18	68	1	Left	Right	No	Sequalae after testis surgery
19	32	1	Left	Bilat	No	Normal appearance
20	53	1	Right	-	No	Bilat varicocele
21	43	2	Bilat	Right	No	Hydrocele (left)
22	29	1	Right	Right	No	Spermatocele (right)
23	56	1	Left	-	No	Hydrocele (left) varicocele (left)
24	64	1	Right	Bilat	No	Varicocele (right)
25	17	1	Left	-	No	Calcification in tunica vaginalis
26	48	1	Right	Right	No	Varicocele (right), spermatocele (left)
27	28	1	Left	-	No	Spermatocele (left), hydrocele (left)
28	50	1	Right	Bilat	No	Kidney lipoma (right)
29	70	1	Left	-	No	Spermatocele (right)
30	23	1	Right	Left	Mixed tumor ^2^	tumor (right)
31	51	Few	Bilat	-	No	Varicocele (right)
32	35	1	Right	-	No	Hydrocele (right)
33	78	Few	Left	Left	No	Hydrocele (bilat), spermatocele (left)
34	57	Few	Left	Bilat	No	Bilat varicocele, bilat atrophic testicles
35	22	Few	Bilat	-	No	Hydrocele (left)
36	23	Few	Right	Right	Embryonal carcinoma	Lesion or tumor (right)
37	46	Few	Left	-	Embryonal carcinoma	Lesion or tumor (sin)
38	28	1	Right	Bilat	no	Spermatocele (left), cyst (right)
39	39	1	Left	-	no	Spermatocele (right)
40	61	1	Left	-	No	Sequalae after vasectomy,
41	41	1	Right	-	Seminoma	Spermatocele (right), cyst (right)
42	36	1	Right	-	No	Lesion (right), varicocele (left)

^1^ Mixed tumor (embryonal carcinoma, seminoma, teratoma, and yolk sac). ^2^ Mixed tumor (teratoma and embryonal carcinoma).

## Data Availability

Data will be available upon request if applicable with Danish legislative.
